# Beyond Landmark Error: Agreement Between AI-Assisted and Manual Cephalometric Tracing at the Level of Clinical Measurements: A Systematic Review

**DOI:** 10.3390/tomography12100146

**Published:** 2026-10-02

**Authors:** Hassan Ahmed Assiri, Huda Alnami, Anwar Abdullah Alsaeed, Hana Almujthel, Zayed Assiri, Mohammed Alalmai

**Affiliations:** 1 Department of Diagnostic Sciences and Oral Biology and Periodontology, College of Dentistry, King Khalid University, Abha 61421, Saudi Arabia; 2 Internship Program, College of Dentistry, King Khalid University, Abha 61421, Saudi Arabia; 3 Department of Oral and Maxillofacial Surgery, Asir Central Hospital, Abha 62411, Saudi Arabia

**Keywords:** artificial intelligence, cephalometry, lateral cephalogram, automated landmark detection, manual tracing

## Abstract

**Background:** Artificial intelligence has revolutionized the field of dentistry. This systematic review evaluated the clinical performance of artificial intelligence (AI)-assisted cephalometric tracing compared with manual or expert digital tracing. **Methods:** We searched the PubMed/MEDLINE, Scopus, and Web of Science databases from inception to 20 May 2026. Studies directly comparing AI-assisted tracing with manual or expert digital tracing of the same lateral cephalograms and reporting at least one clinically relevant cephalometric outcome were included in the review. Two authors independently screened records. Risk of bias was assessed using an adapted QUADAS-2 framework. A quantitative meta-analysis was planned but not performed. Findings were synthesised narratively by measurement, platform, and workflow, with reporting informed by the Synthesis Without Meta-analysis (SWiM) guideline. **Results:** Twenty studies were included. Direct AI-versus-reference ICCs were reported in more than three studies, but their formulations and reference procedures varied. Small average differences sometimes coexisted with wide individual limits of agreement. The numerical evidence did not establish a consistent ranking of SNA, SNB, and ANB. **Conclusions:** AI-assisted tracing may be useful as a clinician-verified adjunct, but its performance depends on the measurement, platform, and workflow. The evidence does not establish uniform clinical interchangeability with manual or expert digital tracing.

## Simple Summary

Orthodontists use head X-rays to measure the teeth and jaws when planning treatment. We reviewed 20 studies comparing measurements produced by artificial intelligence with those made by clinicians. Results varied across software programs and measurements. Small differences on average sometimes hid larger errors in individual patients. Checking and correcting automatically marked points reduced some errors but did not eliminate all differences. Variation in study methods and incomplete reporting limited firm conclusions. Artificial intelligence may assist orthodontic assessment, but its measurements still need professional checking. Future studies should clearly define acceptable errors and use consistent comparison methods.

## 1. Introduction

Since its introduction in 1931, the lateral cephalometric (LC) radiograph has been a fundamental diagnostic imaging tool for the diagnosis and treatment planning of orthodontic cases. It provides a standardised two-dimensional (2D) map of the craniofacial complex, which guides orthodontists accurately [1]. Thus, the LC radiograph is primarily used to assess a patient’s progressive response to treatment and provide a comprehensive view of the relationship between the patient’s skeletal, dental, and soft tissue morphology [1]. In cephalometric analyses, the orthodontist needs to manually detect anatomical landmarks, and this process is time-consuming. In addition, some variability may appear in the results because of image quality and observer skills. In recent years, artificial intelligence (AI) has become helpful in improving diagnostic accuracy, automating analyses, and reducing errors and measurement bias, which improves treatment outcomes. Furthermore, AI can detect cephalometric landmarks in less than one minute. It can also determine which landmarks require more time to identify and which are identified incorrectly. However, the efficiency of AI systems has not yet reached the level of an expert because of the complexity of datasets influenced by factors such as age, gender, size and shape of anatomical structures, class of malocclusion, ethnicity, and bone density. Greater dataset complexity makes AI learning and prediction more challenging, which may increase analytical errors and consequently lead to inaccuracies in diagnosis and treatment planning [2,3,4]. While AI has demonstrated promising capabilities in cephalometric landmark identification, the clinically relevant question is not whether AI can function independently but whether its performance is comparable to that of experienced clinicians. Recent comparative studies have shown that the accuracy of AI-based cephalometric analysis varies considerably across different software platforms and measurement parameters. Research conducted by Bor et al. noted statistically significant differences in several of the main angular and linear measurements produced by AI-assisted tracing software, as compared with those created through an orthodontist performing a manual digital tracing process. The research also indicated that these differences may be due to the methods used to generate these measurements, which can ultimately affect diagnostic conclusions [5]. Additionally, Baig et al. reported similar findings when they evaluated commercially developed AI-based tracing software, which produced measurements that differed from expert-created measurements and was unreliable in measuring some of the most important cephalometric parameters [2]. Besides comparing AI-based tracing with expert-created measurements, Mercier et al. examined the efficiency of completely automated and semi-automated AI systems. They found that, although complete automation significantly reduced the time required to perform the analysis, semi-automation achieved higher levels of accuracy and was more closely aligned with traditional digital tracing results because clinicians could verify and correct the data [6]. These findings suggest that clinician involvement remains essential and that corrected or semi-automated AI workflows may currently offer greater clinical reliability than fully automated analyses alone. Previous reviews have examined both landmark localisation and derived clinical measurements [7,8,9]. Polizzi et al. reviewed fully automatic linear and angular measurements [7], and Narkhede et al. compared digital with manual tracing using meta-analysis [8]. The present review therefore provides an incremental update, rather than the first synthesis of clinical cephalometric measurements. It focuses on direct AI–human comparisons and distinguishes inter-method agreement from examiner reliability, software repeatability, and clinician-corrected workflows. Its additional contribution is the measurement- and platform-specific numerical synthesis of the 20 included studies, not heterogeneity itself.

## 2. Materials and Methods

### 2.1. Study Protocol and Registration

This systematic review was conducted in accordance with the Preferred Reporting Items for Systematic Reviews and Meta-Analyses (PRISMA) guidelines 2020. The protocol was registered with INPLASY (INPLASY202660101) on 21 June 2026, at the data-extraction stage [10]. Registration followed the searches conducted on 20 May 2026 and was not prospective to study selection. The registered strategy allowed narrative synthesis and meta-analysis of compatible estimates. No meta-analysis was performed. During revision, study-level numerical reporting was expanded and the grouping and interpretation of the narrative synthesis were clarified. The original eligibility criteria and 20-study synthesis set were retained.

### 2.2. Search Question

The research question was based on PICO elements and formulated as follows: ‘What is the performance of AI-assisted cephalometric analysis compared with conventional methods?’

Accordingly, PICO elements were as follows:

Population Patients indicated for cephalometry.

Intervention AI-assisted cephalometry.

Comparator Conventional approaches, including human experts’ performance in cephalometry.

Outcome Performance of AI-assisted cephalometry.

### 2.3. Eligibility Criteria

The inclusion criteria comprised studies that reported subjects who had standard LC X-rays or digital lateral cephalograms, along with a direct comparison of AI-assisted cephalometric analysis versus manual/traditional digital tracing by an expert, as well as studies that reported clinically relevant cephalometric output(s), including but not limited to angular/linear measurements, diagnostic category, agreement metrics, reproducibility metrics, and time outcomes. On the other hand, studies were excluded from the primary clinical review if their main purpose was to assess the performance of reduced-field prediction of missing cephalometric values, education/student training, single-image reproducibility without patient-level diagnostic comparison, or landmark detection without clinically relevant cephalometric output.

### 2.4. Search Strategy

**Figure 1:**
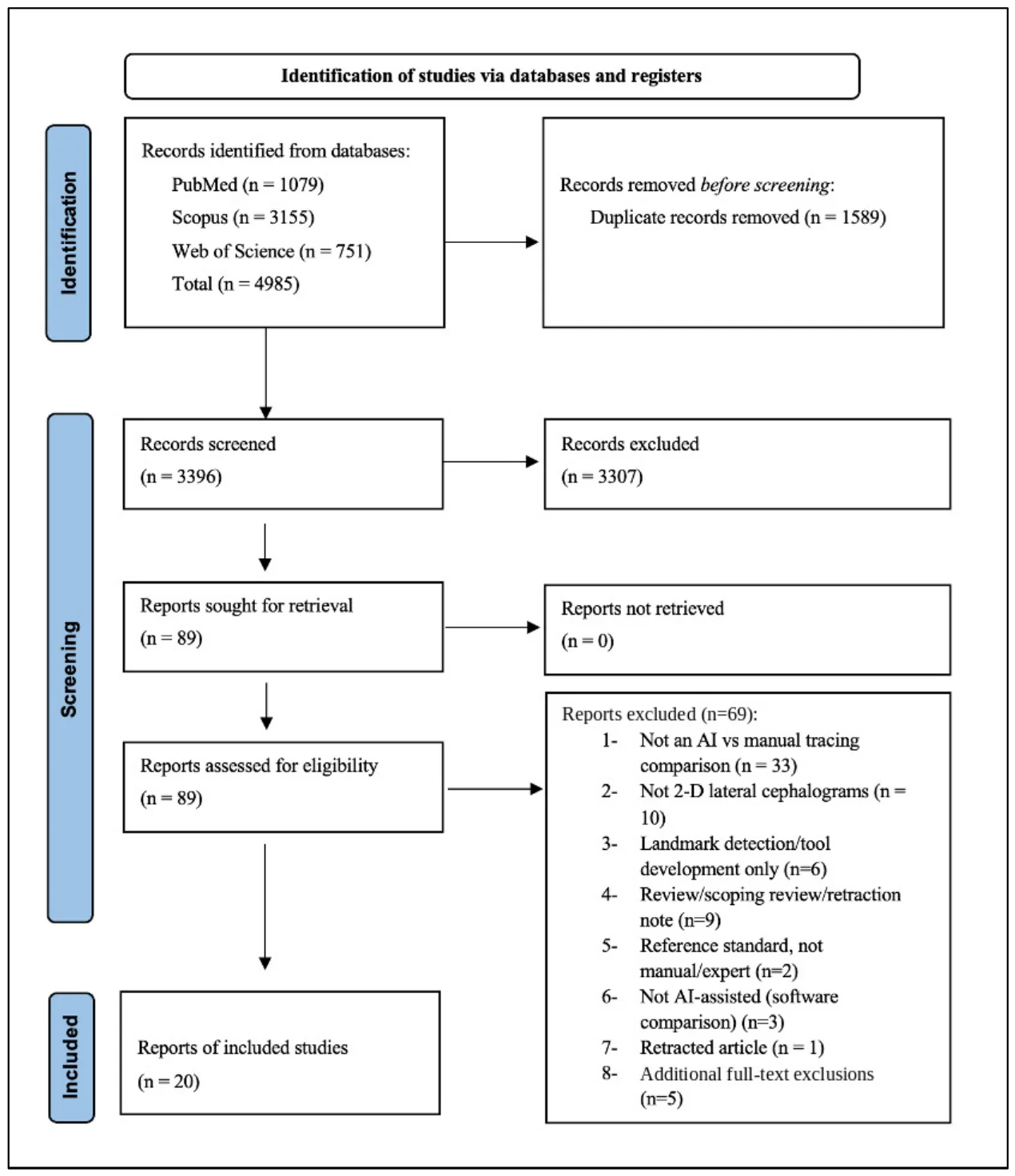
Flowchart of PRISMA.

The searches of PubMed/MEDLINE, Scopus, and Web of Science were conducted on 20 May 2026, covering records from database inception. The search strategy for each database is detailed in Table 1. All retrieved records were uploaded to Rayyan^®^, where duplicates were identified and removed. Two authors independently assessed the titles and abstracts against the eligibility criteria. After that, the same two authors screened the full texts of the relevant studies, and any uncertainty was resolved through group discussion with the senior author. The reviewers then determined the studies suitable for data extraction and quality assessment. This process followed the PRISMA flowchart (Figure 1).

### 2.5. Data Extraction

Data were extracted for study design, population, sample size, AI platform, human reference, cephalometric measurements, and reported statistical comparisons. Direct AI–reference agreement was distinguished from examiner reliability and software repeatability. Fully automatic and clinician-corrected outputs were retained separately. Signed mean differences, mean absolute errors, confidence intervals, Bland–Altman limits of agreement, ICC formulations, and numerical clinical acceptability criteria were recorded where available. Table 2 and Table 4 identify the statistical meaning and source of the results. Missing numerical information was not imputed or interpreted as evidence of acceptable agreement.

### 2.6. Quality Assessment

**Figure 2:**
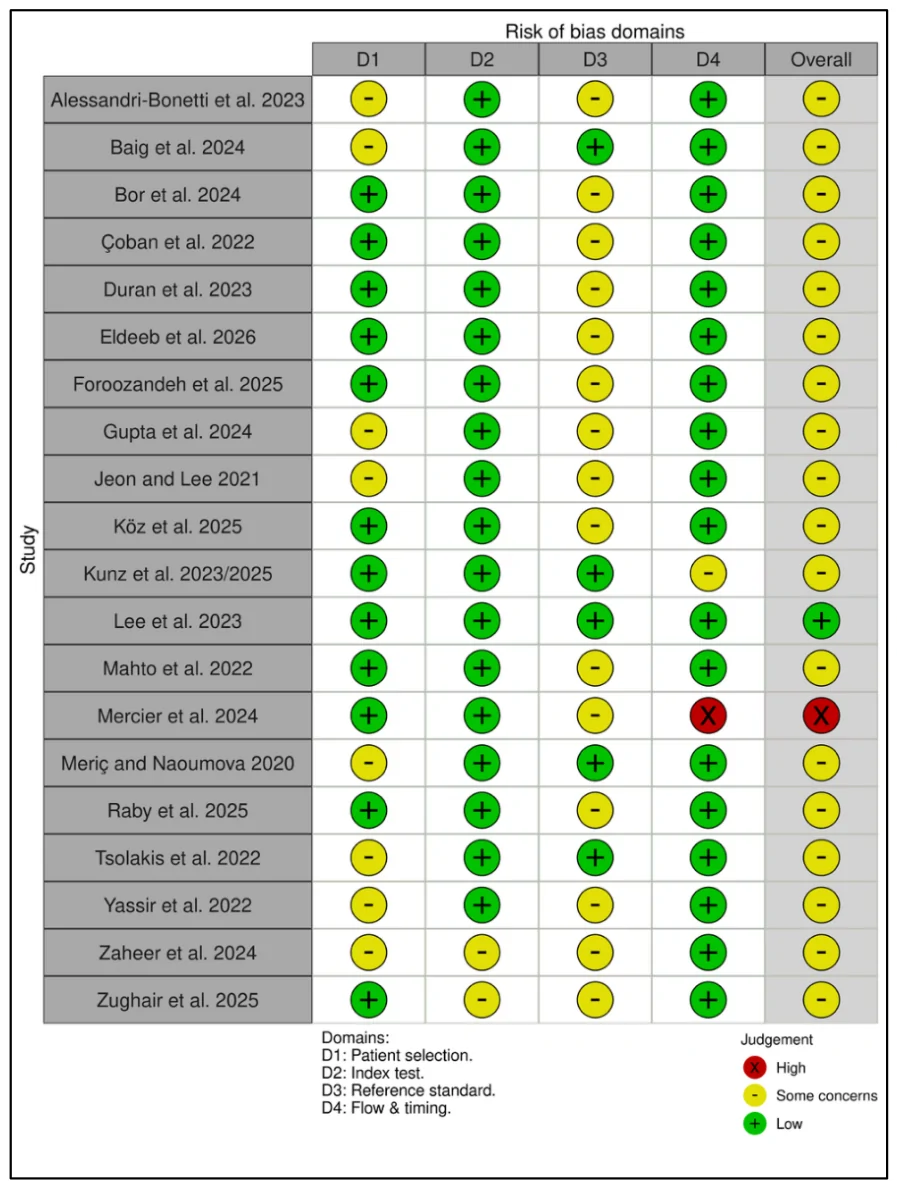
Traffic-light display of the adapted QUADAS-2 risk-of-bias assessment for the 20 included studies. Domain and overall judgments correspond to Table 3. Each row represents one of the 20 included studies. Green denotes low risk, yellow denotes unclear risk (labelled “some concerns” in the graphic), and red denotes high risk. D1, patient selection; D2, index test; D3, reference standard; D4, flow and timing. The domain and overall risk-of-bias ratings correspond to Table 3. These ratings are not certainty-of-evidence grades [2,5,6,13,14,15,16,17,18,19,20,21,22,23,24,25,26,27,28,29].

Risk of bias was assessed using an adapted QUADAS-2 framework covering patient selection, the AI index test, the human reference standard, and flow and timing [11]. The framework was used because these studies compared paired image-based measurements with a human reference, rather than primarily developing or validating multivariable clinical prediction models. PROBAST addresses prediction-model risk of bias and was not used as a substitute for appraisal of measurement agreement [12]. The QUADAS-2 adaptation is not a validated instrument for continuous method-comparison studies. Patient-selection judgments considered recruitment, sampling, and exclusions, rather than sample size alone. Index-test judgments considered whether automatic outputs or clinician corrections were obtained independently of the reference. Reference-standard judgments considered examiner expertise, independent tracing, repeatability, and consensus procedures; a single examiner was not automatically judged to create high risk. Flow-and-timing judgments considered whether all selected images received both measurements and whether failures or exclusions depended on AI performance. Each domain was rated low, unclear, or high risk; overall risk was high if any domain was high, low only if all domains were low, and unclear otherwise. Applicability was considered separately. Two reviewers assessed the studies independently and resolved disagreements through discussion, as specified in the original Methods and registered protocol [10]. The screening process also involved two authors. Table 3 presents the corrected judgments, with their reasons explained below. Figure 2 displays the same risk-of-bias judgments.

**Table 2:** Data extraction of the retrieved studies.

Author & Year	Country	Design/Objective	Sample	AI Index Test	Reference Standard	Measurements (Key Params)	Statistics
Alessandri-Bonetti et al. 2023 [13]	Italy	Retrospective pilot method-comparison/reliability study; tested the reliability of AI-assisted cephalometric analysis versus manual digital analysis.	13 lateral cephalograms; 9 females/4 males; mean age 20.0 ± 17.5 years (range 6.3–65.9).	Novarad^®^ fully automated cephalometric analysis performed in a specialised radiology laboratory.	An experienced orthodontist used Openceph v4.1.0; manual landmarking repeated twice, with a two-week interval. The second tracing served as the reference for inter-method comparison.	25 landmarks; 5 Ricketts parameters: maxillary convexity, facial conicity angle, facial axis angle, posterior facial height, and lower facial height.	Shapiro–Wilk, Dahlberg error, Bland–Altman analysis.
Baig et al. 2024 [2]	United Arab Emirates	Retrospective comparative diagnostic/concordance study of three commercial AI cephalometric programs against human expert tracing.	63 pretreatment lateral cephalograms from patients with late mixed/permanent dentition; excluded cleft lip/palate, asymmetry, previous orthodontic treatment, and artefacts.	WebCeph™, Cephio, and Ceppro DDH Inc.; fully automated tracing/analysis with no human input. Ten radiographs re-uploaded after one week to assess AI reliability.	Dolphin Imaging semi-automatic tracing performed by a resident, verified/adjusted by an orthodontist with >15 years’ experience; a second expert traced as well; the median of both served as the reference.	11 parameters including SNA, SNB, ANB, *Y*-axis, FMA, SN-MP, U1-NA, U1-APog, L1-MP, interincisal angle, and nasolabial angle; diagnostic sensitivity/specificity; and time.	ICC for human intra/inter-examiner reliability and AI repeatability; repeated-measures ANOVA with Bonferroni comparisons; diagnostic sensitivity/specificity; time.
Bor et al. 2024 [5]	Turkey	Retrospective class-stratified method-comparison study comparing AI-assisted cephalometry with orthodontist-performed digital tracing.	120 lateral cephalograms randomly selected from 1500 eligible records: 40 Class I, 40 Class II, and 40 Class III; age 12–18 years.	WebCeph v1.5.0, WeDoCeph, and CephX; fully automated landmarking/measurements without manual intervention. WebCeph/WeDoCeph manually calibrated; CephX AI calibrated.	NemoCeph digital tracing performed under the final decision of an experienced orthodontist, with assistance from five final-year residents.	7 angular parameters (ANB, SNB, SNA, FMA, IMPA, NLA, and U1/SN) and 7 linear parameters (U1/NA, L1/NB, Co-A, ANS-Me, Co-Gn, U-E line, and L-E line).	ANOVA/Tukey comparisons; human intra/inter-rater ICCs; repeated AI outputs assessed separately. Human reliability ICCs do not quantify AI–reference agreement.
Çoban et al. 2022 [14]	Turkey	Comparative study of digital manual tracing versus web-based AI tracing, stratified by sagittal skeletal malocclusion.	105 individuals; 49 females/56 males; mean age 17.25 ± 1.87 years; 35 Class I, 35 Class II, and 35 Class III.	WebCeph automatic web-based AI cephalometric tracing.	Dolphin Imaging v11.5 digital manual tracing performed by a single experienced orthodontist.	10 linear and 12 angular measurements, including SNA, SNB, ANB, Wits-related/NA/NPog, SN.GoGn, FMA-related, incisor, E-line, and nasolabial angle measurements.	Paired *t*-tests, ANOVA, and pairwise manual–AI ICCs; separate repeatability assessment on 10 images.
Duran et al. 2023 [15]	Turkey	Retrospective archive-based comparison of fully automatic AI cephalometric software versus non-automated digital tracing.	526 radiographs screened; 336 eligible after exclusions; 50 randomly selected; patients aged 12–20 years.	OrthoDx™ and WebCeph fully automatic AI analyses.	Dolphin 11.8 manual landmarking performed by an orthodontist with 4 years’ experience; two investigators assessed reference reliability.	32 landmarks; 11 angular, 4 linear, 1 proportional, and 1 soft-tissue measurement from Steiner/Jarabak-style analyses.	Direct Dolphin–AI ICCs and signed differences with paired SDs in Tables 1–2; separate examiner ICCs in Table 3. ICC model/form/unit unspecified. One-sample tests of differences; Bland–Altman plots for selected non-sagittal measurements.
Eldeeb et al. 2026 [16]	Egypt	Comparative in silico study evaluating a web-based AI platform and Carestream AI versus manual digital tracing.	57 pretreatment lateral cephalograms from Alexandria University archives; Class I/II/III included; excluded dentofacial disorders, congenital abnormalities, mixed dentition, poor-quality images, and artefacts.	WebCeph and Carestream Imaging Software V8 AI; fully automated after calibration with no manual correction.	OnyxCeph3 manual digital tracing performed by one radiologist, validated/calibrated by an experienced orthodontist; served as the control/gold standard.	18 measurements: 5 linear, 12 angular, and 1 ratio; 11 skeletal and 7 dental; Steiner and Tweed analyses.	Repeated-measures ANOVA/Friedman with Bonferroni comparisons; mean differences and 95% CIs in Tables 5–6. Table 2 reports examiner reliability. Between-method ICCs are located in a separate supplement; the main-text ICC ranges are reported in Table 4.
Foroozandeh et al. 2025 [17]	Iran	Cross-sectional comparative accuracy/reliability study of AI-based AudaxCeph, Dolphin, and manual tracing.	60 lateral cephalograms randomly retrieved from 2021–2022 records; permanent dentition; good image quality.	AudaxCeph automated AI mode and AudaxCeph semi-automated mode with manual landmark correction.	Manual tracing served as the gold standard, alongside Dolphin software; observers included a trained final-year dental student and an oral/maxillofacial radiologist, supervised by an orthodontist with >15 years’ experience.	23 landmarks; 10 indices: GoGn-SN, SNA, SNB, ANB, interincisal angle, U1-NA degrees/mm, L1-NB degrees/mm, lower lip to E-line.	Three-method, average-measures (k = 3), two-way random, absolute-agreement ICCs; Bland–Altman plots; separate inter/intra-observer reliability.
Gupta et al. 2024 [18]	India	Cross-sectional comparison of smartphone app-based and AI web-based cephalometric tracing versus manual tracing, including speed.	40 lateral cephalograms from adults aged 18–40 years; records from January–June 2022.	WebCeph v1.0 fully automated without correction and WebCeph with manual landmark correction; OneCeph smartphone app also tested, although app-based rather than fully AI.	Manual tracing with a pencil/ruler/protractor; measurements performed by the principal investigator.	SNA, SNB, ANB, SN-GoGn, UI-NA degrees/mm, LI-NB degrees/mm, occlusal plane-SN, upper/lower lip to E-line, and analysis time.	Omnibus ICC across four methods; paired *t*-tests with mean differences and paired SDs. The omnibus coefficient is not isolated AI–manual agreement.
Jeon and Lee 2021 [19]	South Korea	Retrospective comparison of conventional cephalometric analysis versus automatic CNN-based cephalometric analysis.	35 orthodontic patients; 20 men/15 women; mean age 23.8 years; fully erupted permanent dentition.	CephX™ (ORCA Dental AI), convolutional neural network-based automatic cephalometric analysis.	Conventional analysis with V-ceph™ v8.0.	16 landmarks; 15 skeletal, 9 dental, and 2 soft-tissue measurements.	Paired *t*-tests and Bland–Altman plots; sample-size calculation reported.
Köz et al. 2025 [20]	Turkey	Retrospective comparative reliability and speed study comparing AI-supported WebCeph and app-aided OneCeph versus manual tracing.	110 randomly selected from 400 eligible records; 47 males/63 females; mean age 14.6 years (range 10–32); permanent dentition.	WebCeph AI-supported automatic analysis. OneCeph app also evaluated, although app-aided rather than fully AI.	Manual acetate/radiographic printout tracing performed by an author with 3 years’ experience and verified by an author with 20 years’ experience.	10 skeletal, 6 dental, and 5 soft-tissue landmarks; 11 skeletal, 6 dental, and 3 soft-tissue measurements; analysis time.	Pairwise manual–WebCeph ICCs with CIs; separate repeatability ICCs on 30 images after one month; Kruskal–Wallis test for time.
Kunz et al. 2025 [21]	Germany	Multi-provider comparison of four commercial AI cephalometric systems versus a human orthodontic expert gold standard.	50 randomly selected cephalometric radiographs from a pool of 3000; 3 excluded because of artefacts; approximately 47 analysed.	DentaliQ.ortho, WebCeph, AudaxCeph, and CephX; fully automated analyses repeated after four months.	Human gold standard defined as the median of 12 experienced orthodontic examiners (6 specialists and 6 advanced postgraduate residents).	15 landmarks; 9 parameters: SNA, SNB, ANB, SN-PP, SN-MeGo, PP-MeGo, facial height ratio, U1-SN, and L1-MeGo.	Repeated-measures ANOVA/Bonferroni; Bland–Altman bias and limits; separate trueness and precision criteria. ICCs concern human reference reliability.
Lee et al. 2023 [22]	South Korea	Retrospective comparison of four commercial AI systems versus manual expert landmarking, with emphasis on interchangeability and diagnostic classification reliability.	84 lateral cephalograms; 46 males/38 females; mean age 11.13 ± 3.52 years; Class I *n* = 35, Class II *n* = 24, and Class III *n* = 25.	CellmatIQ, CephX, AudaxCeph Automatic Tracing Mode 6.3, and WebCeph 1.0.0 without corrections.	Two board-certified orthodontists (>7 and >5 years’ experience) manually landmarked with AudaxCeph Sequential Tracing; expert 1 served as the main reference, and expert 2 assessed clinical acceptability/inter-examiner error.	15 landmarks; 13 variables, including SNA, SNB, ANB, Wits, FMA, SN-MP, Björk-Jarabak sum, SN-U1, FH-U1, IMPA, U1L1, SN-OcP, and FH-OcP.	Bland–Altman bias/limits and human-error-based interchangeability; pairwise absolute-agreement and consistency ICCs; separate examiner reliability.
Mahto et al. 2022 [23]	Nepal	Comparative validity and reliability study of fully automated WebCeph versus manual cephalometric tracing.	30 pretreatment lateral cephalograms; 8 males/22 females; mean age 20.17 ± 6.72 years.	WebCeph fully automated AI, calibrated with a 10 mm ruler.	Manual tracing on printed digital images performed by an orthodontist with >10 years’ experience; a maximum of 5 images/day to reduce fatigue.	18 landmarks; 12 measurements, including SNA, SNB, ANB, S-N to Go-Gn, FMA, U1-NA degrees/mm, L1-NB degrees/mm, IMPA/L1-MP, and upper/lower lip to E-line.	Pairwise manual–WebCeph ICCs with CIs; separate repeatability ICCs on 10 images after 10 days.
Mercier et al. 2024 [6]	Spain	Diagnostic accuracy/method-comparison study following STARD; compared manual, fully automatic AI, and semi-automatic AI cephalometric analysis.	408 lateral cephalograms after 25 exclusions; 167 males/241 females; skeletal Class I 34.6%, Class II 56.1%, and Class III 9.3%.	WebCeph fully automatic and WebCeph semi-automatic mode with manual landmark adjustment.	NemoCeph conventional digital manual technique served as the reference; the main operator completed the analyses, with reliability testing on 10% of the sample against a second operator/professor.	15 variables from Steiner, Jarabak, and Ricketts analyses, including SNA, SNB, ANB, SN-MP, Wits, U1-NA, L1-NB, U1/L1-APg, IMPA, and lower lip E-plane.	Signed mean differences (trueness), SEs and CIs; paired tests; time comparisons; human intra/inter-operator ICCs, not AI–reference ICCs.
Meriç and Naoumova 2020 [24]	Turkey	Comparative study of fully automated, corrected automated, app-aided, computerised, and manual cephalometric tracing, including analysis time.	40 pretreatment lateral cephalograms; 7 males/33 females; mean age 16.0 ± 4.6 years.	CephX fully automated AI, with a corrected CephX arm after manual landmark correction. CephNinja app and Dolphin also compared.	Manual ground truth defined as the mean of three manual observers; the same operator completed the digital/app/AI tracings.	8 angular and 4 linear parameters; skeletal, dental, and soft-tissue measurements; analysis time.	ANOVA/Tukey comparisons; within-method repeatability ICCs and manual inter-observer ICCs, not pairwise AI–manual ICCs.
Raby et al. 2025 [25]	Chile	Comparative accuracy, reliability, and time-efficiency study of two AI web platforms in automated and corrected forms versus Dolphin.	50 pretreatment digital lateral cephalograms from patients attending Universidad de los Andes between 2019 and 2023.	WebCeph and CephX in automated form and landmark-corrected form.	Dolphin Imaging v13.01 served as the gold standard; single examiner with 5 years’ Dolphin and 2 years’ AI-platform experience.	12 parameters: ANB, SNA, SNB, facial convexity, SNGoGn, facial axis, PP-PM, U1-APo, L1-APo, U1-PP, IMPA, NLA, and tracing time.	OLS software comparisons; direct Dolphin–platform ICCs in Table IV, with one unassigned CI column per parameter. Separate 15-image calibration ICC 0.994. Clinical criterion >2°/>2 mm; corrected and automatic outputs analysed separately.
Tsolakis et al. 2022 [26]	United States/private practice setting	Method-comparison study comparing fully automated CS Imaging V8 tracing versus manual digital Dolphin tracing for orthodontic diagnosis.	100 pretreatment lateral cephalograms; 43 males/57 females; mean age 15.9 ± 4.8 years.	CS Imaging V8 fully automatic digital tracing.	Dolphin 3D Imaging v11.0 manual digital tracing performed by a blinded, experienced operator who was a Diplomate of the American Board of Orthodontics.	ABO and EBO cephalometric measurements; 16 landmarks; the methods section reported 17 angular and 2 linear measurements.	Pairwise manual–AI ICCs; paired *t*-test/Wilcoxon with multiplicity adjustment; separate intra-method repeatability.
Yassir et al. 2022 [27]	Iraq	Accuracy and reliability study comparing automatic and semi-automatic WebCeph versus AutoCAD as the reference standard.	50 pretreatment lateral cephalograms; 25 females/25 males; skeletal Class I *n* = 30, Class II *n* = 10, Class III *n* = 10.	WebCeph automatic and WebCeph semi-automatic options.	AutoCAD software served as the reference standard, with manual landmark identification; the investigator received training from an expert orthodontist; 10 radiographs used to assess inter/intra-examiner reliability.	17 landmarks; 11 measurements: SNA, SNB, ANB, SN-MP, U1-MaxP, L1-MP, N-Me, U1-NPog, L1-NPog, upper lip E-line, and lower lip E-line.	Pairwise AI–reference ICCs, paired mean-difference CIs, Bland–Altman plots and proportional-bias regression; examiner reliability assessed separately.
Zaheer et al. 2024 [28]	Pakistan	Comparative study of manual, semi-automatic WebCeph, and fully automatic CephX cephalometric tracing and evaluation.	54 participants; mean age 16.7 ± 1.6 years; median 15.5 years; 21 males/33 females.	WebCeph semi-automatic and CephX fully automatic AI-driven software.	Manual tracing; two investigators assessed each of the 18 parameters with all three methods.	18 variables, including SNA, SNB, IMPA, interincisal angle, E-line upper/lower lip, SN-GoGn, *Y*-axis, Jarabak, SNP, MMA, IPP, Wits, NLA, SNO, and McNamara to point A.	Inter-examiner ICCs within methods and paired examiner tests; ANOVA/post-hoc between-method comparisons. These ICCs are not AI–manual agreement.
Zughair et al. 2025 [29]	Yemen	Retrospective cross-sectional analytical study comparing AI-based automatic tracing, semi-automatic digital tracing, and manual digital tracing.	120 pretreatment lateral cephalograms from Sana’a University Orthodontic Clinic; 71 females/49 males; mean age 20.3 ± 3.9 years (range 14–33).	AI-based automatic digital tracing and semi-automatic digital tracing with manual corrections by orthodontic specialists. WebCeph is discussed as the example/program context; manual tracing performed with OneCeph.	Manual digital tracing served as the control; orthodontic residents calibrated by an expert orthodontist; expert/resident inter- and intra-examiner reliability assessed with OneCeph.	34 measurements derived from 20 skeletal and 10 soft-tissue landmarks; extracted parameters included SNA, A-N, SNB, MP-SN, Pg-N, ANB, Go-Gn-SN, interincisal, L1-NB, U1-NA, and nasolabial angle.	Repeated-measures ANOVA/Bonferroni and Friedman/Dunn tests; examiner calibration described, without numerical inter-method ICCs.

**Table 3:** Methodological quality and risk of bias of the included studies according to the modified QUADAS-2 tool.

Study	Patient Selection	Index Test	Reference Standard	Flow and Timing	Overall RoB	Overall Applicability
Alessandri-Bonetti et al. (2023) [13]	U	L	U	L	U	U
Baig et al. (2024) [2]	U	L	L	L	U	L
Bor et al. (2024) [5]	L	L	U	L	U	U
Çoban et al. (2022) [14]	L	L	U	L	U	U
Duran et al. (2023) [15]	L	L	U	L	U	U
Eldeeb et al. (2026) [16]	L	L	U	L	U	L
Foroozandeh et al. (2025) [17]	L	L	U	L	U	U
Gupta et al. (2024) [18]	U	L	U	L	U	U
Jeon and Lee (2021) [19]	U	L	U	L	U	U
Köz et al. (2025) [20]	L	L	U	L	U	H
Kunz et al. (2023) [21]	L	L	L	U	U	U
Lee et al. (2023) [22]	L	L	L	L	L	U
Mahto et al. (2022) [23]	L	L	U	L	U	U
Mercier et al. (2024) [6]	L	L	U	H	H	L
Meriç and Naoumova (2020) [24]	U	L	L	L	U	U
Raby et al. (2025) [25]	L	L	U	L	U	U
Tsolakis et al. (2022) [26]	U	L	L	L	U	U
Yassir et al. (2022) [27]	U	L	U	L	U	U
Zaheer et al. (2024) [28]	U	U	U	L	U	U
Zughair et al. (2025) [29]	L	U	U	L	U	U

L, low risk of bias or low applicability concern; U, unclear risk of bias or applicability; H, high risk of bias or applicability. QUADAS-2, Quality Assessment of Diagnostic Accuracy Studies, version 2. The framework was adapted for paired AI-assisted and manual or expert digital cephalometric measurements. Overall risk was high if any domain was high, low only if all domains were low, and unclear otherwise. Applicability was assessed separately. These judgments are not formal certainty-of-evidence grades.

### 2.7. Evidence Certainty

No formal certainty-of-evidence framework was applied. Interpretation considered recruitment and exclusion procedures, reference-standard limitations, imprecision, inconsistency across measurements and software, and incomplete numerical reporting. Domain-level risk-of-bias judgments were not converted into a certainty rating for the body of evidence.

### 2.8. Data Synthesis

No quantitative meta-analysis was performed. The narrative synthesis was organised by clinical measurement, AI platform, human reference, and fully automatic versus clinician-corrected workflow. Multiple software or correction arms using the same images were retained as separate comparisons but not counted as independent samples. Direct AI–reference ICCs were separated from examiner reliability, software repeatability, and coefficients combining several methods. The ICC model, absolute-agreement or consistency definition, and single- or average-measure unit were recorded when reported [30]. Signed mean differences, mean absolute errors, and Bland–Altman limits of agreement were considered separately. Numerical estimates, uncertainty, and the original studies’ clinical margins took priority over significance tests or qualitative labels. Conflicting findings were interpreted within their population, platform, reference, workflow, and risk-of-bias context; no significance-based vote counting or statistical weighting was used. A clinical margin not defined by the source was recorded as absent. During revision, mean-difference pooling was considered separately from ICC pooling. Paired standard deviations were available in Çoban, Duran, Gupta, and Jeon and Lee, and mean-bias confidence intervals were available in other reports [6,14,15,16,18,19,21,22,27]. A restricted synthesis of average bias was therefore feasible in principle. It was not undertaken because an average-bias estimate alone would not answer the principal question of individual clinical interchangeability, and compatible individual-agreement estimates and clinical margins were not consistently available. This was a decision not to implement the protocol’s conditional mean-difference analysis, not evidence that all quantitative synthesis was impossible. Reporting followed SWiM where applicable [31]. No pooled agreement estimate, equivalence estimate, or statistical ranking of SNA, SNB, and ANB was calculated. Descriptive analyses were performed in Stata 19/BE (StataCorp, 2025. Stata Statistical Software: Release 19. College Station, TX, USA: StataCorp LLC).

## 3. Results

The searches identified 4985 records: PubMed/MEDLINE, 1079; Scopus, 3155; and Web of Science, 751. Records were uploaded to Rayyan for deduplication. After removal of 1589 duplicates, two authors screened 3396 records and excluded 3307. The same two authors assessed 89 full-text reports. The recorded exclusions comprised 64 reports at the initial full-text stage and five additional reports at detailed full-text assessment, giving 69 excluded reports and 20 included studies. Table S1 provides the original reasons for these five decisions [32,33,34,35,36]. Lee Part 2 was excluded to avoid duplicate use of the 84-cephalogram cohort already represented by Part 1 [22,35]. Panesar was excluded because its ICCs assessed intra-rater precision and its gold-standard comparison used mean absolute error rather than a poolable AI–reference ICC [36]. These were not exclusions based on QUADAS-2 ratings. Figure 1 presents the reconciled counts.

### 3.1. Risk of Bias Assessment

Patient selection was rated low risk in 12 studies and unclear in eight. The index-test domain was low risk in 18 studies and unclear in two; the reference-standard domain was low risk in five and unclear in 15. Flow and timing were low risk in 18 studies, unclear in one, and high risk in one. Overall, one study was low risk, 18 were unclear, and one was high risk. Applicability concerns were predominantly unclear: 16 studies were unclear, three low, and one high. Table 3 reports these judgments, and Figure 2 displays the same domain and overall risk-of-bias ratings.

The judgments concern recruitment and exclusions, not sample size alone. In Alessandri-Bonetti et al., the sample comprised records with existing AI analyses; selection independent of successful AI processing was unclear, but a sample of 13 was not itself evidence of high selection bias [13]. In Gupta et al., the reporting of recruitment and the reference procedure supported uncertainty rather than a high-risk rating based only on sample size or unstated examiner experience [18]. Kunz et al. randomly selected 50 images from 3000 and excluded three for radiographic artefacts, not documented AI-recognition failures; flow was therefore rated unclear rather than high [21]. Mahto et al. randomly selected eligible images, supporting low selection risk despite the small sample [23]. By contrast, Mercier et al. excluded 25 of 433 images because AI could not recognise or adequately landmark them, supporting high flow-and-timing risk [6]. Duran and Raby used randomly selected clinical images but imposed image-quality or anatomical restrictions; the independence of their single-examiner reference from the AI results was not clearly established [15,25]. Eldeeb used random selection, randomised software order, and a blinded statistician, but the tracing operator was not blinded [16]. Unreported independence and restricted patient or image spectra were retained as uncertainty or applicability concerns rather than assumed absent.

### 3.2. Synthesis of Findings

Table 4 reports numerical findings for all 20 included studies, including the additional software arms and clinician-corrected comparisons. Direct AI–reference ICCs were reported by Çoban, Duran, Köz, Lee, Mahto, Raby, Tsolakis, and Yassir [14,15,20,22,23,25,26,27]. Eldeeb also reported between-method ICC results, separately from examiner reliability [16]. Foroozandeh and Gupta reported coefficients combining several methods; these do not estimate a single AI–manual pair [17,18]. For SNA, SNB, and ANB, respectively, Köz reported ICCs of 0.692 (95% CI 0.390–0.826), 0.883 (0.829–0.920), and 0.845 (0.434–0.934); Mahto reported 0.879 (0.745–0.943), 0.899 (0.770–0.954), and 0.908 (0.747–0.961) [20,23]. Neither study defined a numerical angular acceptability margin. Duran reported corresponding ICCs of 0.750/0.846/0.876 for OrthoDx and 0.736/0.862/0.791 for WebCeph against Dolphin, with signed differences and paired standard deviations reported in Table 4 [15]. Raby reported automatic CephX ICCs of 0.93/0.96/0.95 and automatic WebCeph ICCs of 0.81/0.91/0.83, yet its assessment of individual differences supported SNA and SNB only after landmark correction [25]. Its single confidence-interval column could not be assigned to the four software/workflow estimates and was not treated as four separate intervals. The evidence did not support a uniform ranking of SNB, SNA, and ANB. Baig reported a human-minus-WebCeph SNB difference of −2.3°, exceeding that study’s 2° clinical convention [2]. In Lee, automatic WebCeph SNB bias was −0.24° (95% CI −0.71 to 0.23), but the 95% limits of agreement were −4.48° to 4.00°; none of the four automatic platforms met the study’s interchangeability criteria for all three sagittal angles [22]. A missing clinical threshold or a non-significant test was not interpreted as evidence of equivalence. Eldeeb’s main text reported WebCeph–manual ICCs above 0.8 except for 1u–NA (0.529), and Carestream–manual ICCs of 0.386–0.833 except for anterior facial height (0.970); exact parameter-level between-method values were in a separate supplement that could not be retrieved [16].

## 4. Discussion

This review examined how closely AI-assisted cephalometric tracing reproduces manual or expert digital measurements. The results support a measurement- and platform-specific interpretation, rather than uniformly good agreement. A small mean difference can coexist with wide limits of agreement for individual patients, as illustrated by the sagittal results in Lee et al. [22]. Non-significant ANOVA or paired-test results do not establish equivalence. A consistency ICC may remain high despite systematic bias; an absolute-agreement ICC incorporates disagreement attributable to such bias but also depends on sample variability, model and measurement unit [30]. Neither form alone establishes clinical interchangeability. Signed bias, mean absolute error, confidence intervals, limits of agreement, and a justified clinical margin answer different questions and were not treated as interchangeable outcomes.

Previous reviews have addressed both landmark localisation and clinically derived measurements. Schwendicke et al. and de Queiroz et al. synthesised landmark-detection performance [37,38], while Hendrickx et al. pooled landmark-error data from 27 two-dimensional studies [3]. These landmark-level findings cannot by themselves establish acceptable error for a particular clinical angle. The present review therefore interprets clinical measurements separately, without claiming that earlier syntheses were confined to landmark error.

Polizzi et al. included 10 studies of fully automatic clinical linear and angular measurements and searched through 22 March 2025 [7]. Seven also appear in the present review: Duran, Mahto, Jeon and Lee, Meriç and Naoumova, Yassir, Mercier, and Zaheer. Their remaining three studies were Prince, El-Dawlatly, and Sadek; the present 20-study set contains 13 other studies. Narkhede et al. included 20 studies of digital-versus-manual tracing published from January 2013 to July 2023, with 14 contributing to meta-analysis, including SNA, SNB, and ANB [8]. None of its 20 Table 1 entries is the same report as the 20 studies in the present review; its Mahto report is from 2016, not the 2022 study included here. Narkhede’s review was not restricted to AI. In contrast, our synthesis is confined to direct AI–human clinical comparisons and separates inter-method agreement, repeatability, and corrected workflows. These differences define an incremental contribution; they do not establish that clinical measurement synthesis is new or that this review supersedes the earlier reviews.

Clinician correction reduced some discrepancies but did not establish universal interchangeability. Mercier et al. reported smaller sagittal mean differences after correction, while Yassir et al. reported persistent error for the lower-incisor-to-mandibular-plane angle even after correction [6,27]. Fully automatic and corrected results were therefore reported separately; neither workflow was pooled. The platform- and parameter-specific variability in Baig and Bor also argues against interpreting a single overall accuracy label as evidence that all clinical measurements are acceptable [2,5].

### Strengths and Limitations

Strengths include independent screening by two authors and a numerical synthesis that distinguishes inter-method agreement from repeatability. The search was restricted to PubMed/MEDLINE, Scopus, and Web of Science; missed studies and publication bias cannot be excluded. Registration occurred at the data-extraction stage, after study selection. The adapted QUADAS-2 framework has not been validated specifically for continuous cephalometric agreement studies, and no formal certainty framework was applied. Some agreement statistics were incompletely specified: Duran did not report the ICC model, form, or unit, and Raby provided an unassigned confidence-interval column alongside four software-specific ICCs [15,25]. Eldeeb’s separate between-method ICC supplement was not available for parameter-level extraction [16]. Reference methods, patient spectra, platforms, correction procedures, and clinical margins varied. Although average-bias pooling was feasible in principle for some comparisons, it was not performed; no pooled agreement estimate or formal between-parameter ranking is available. Findings for sagittal angles should not be generalised to all vertical, dental, or soft-tissue measurements.

## 5. Conclusions

AI-assisted cephalometric tracing may be useful as a clinician-verified adjunct, but performance depends on the measurement, platform, and workflow. The available evidence does not establish uniformly acceptable SNA, SNB, or ANB measurements, a consistent ranking of these parameters, or interchangeability for individual patients. Interpretation is limited by heterogeneous methods, reference-standard concerns, incomplete numerical reporting, and the absence of a pooled estimate or formal certainty assessment. AI-assisted analysis should complement rather than replace expert verification. Future studies should use multi-observer or consensus reference standards and report agreement across skeletal, dental, and soft-tissue parameters.

## Figures and Tables

**Table 1:** Search strategies for the databases searched on 20 May 2026.

Database	Search Strategy
PubMed/MEDLINE	(“artificial intelligence”[tiab] OR “machine learning”[tiab] OR “deep learning”[tiab] OR “convolutional neural network”[tiab] OR CNN[tiab] OR automat *[tiab] OR “computer-assisted”[tiab] OR WebCeph[tiab] OR CephX[tiab] OR Ceppro[tiab] OR WeDoCeph[tiab] OR AudaxCeph[tiab] OR “Artificial Intelligence”[Mesh] OR “Machine Learning”[Mesh] OR “Deep Learning”[Mesh]) AND (cephalometr *[tiab] OR “lateral cephalogram *”[tiab] OR “cephalometric analysis”[tiab] OR “cephalometric landmark *”[tiab] OR “cephalometric tracing”[tiab] OR “Cephalometry”[Mesh])
Scopus	TITLE-ABS-KEY (“artificial intelligence” OR “machine learning” OR “deep learning” OR “convolutional neural network” OR CNN OR automat * OR “computer-assisted” OR WebCeph OR CephX OR Ceppro OR WeDoCeph OR AudaxCeph) AND TITLE-ABS-KEY (cephalometr OR “lateral cephalogram *” OR “cephalometric analysis” OR “cephalometric landmark” OR “cephalometric tracing”) AND TITLE-ABS-KEY (accuracy OR accurate OR reliable OR agreement OR validity OR valid OR comparison OR compare OR reproducible OR precision OR performance OR “manual tracing” OR “expert tracing” OR “digital tracing”)
Web of Science Core Collection	TS = (“artificial intelligence” OR “machine learning” OR “deep learning” OR “convolutional neural network” OR CNN OR automat OR “computer-assisted” OR WebCeph OR CephX OR Ceppro OR WeDoCeph OR AudaxCeph) AND TS = (cephalometr OR “lateral cephalogram” OR “cephalometric analysis” OR “cephalometric landmark” OR “cephalometric tracing”) AND TS = (accuracy OR accurate OR reliab OR agreement OR validity OR valid OR comparison OR compare OR reproducible OR precision OR performance OR “manual tracing” OR “expert tracing” OR “digital tracing”)

**Table 4:** Numerical synthesis of the original 20 included studies.

Study and Sample	AI and Human Reference	Measurement-Specific Numerical Findings	Statistical Meaning and Clinical Criterion
Alessandri-Bonetti 2023 [13] *n* = 13	Novarad/OpenCeph	Dahlberg error: convexity 0.519 mm; conicity 0.969°; facial axis 1.854°; posterior height 1.732 mm; lower facial height 1.455°. SNA/SNB/ANB not measured.	Inter-method error, not ICC. The source interval label is ambiguous. No numerical clinical margin was defined.
Baig 2024 [2] *n* = 63	WebCeph, Cephio, Ceppro/expert Dolphin median	Human minus AI, SNA/SNB/ANB: WebCeph −4.7/−2.3/−2.4°; Cephio −0.7/−0.3/−0.4°; Ceppro +0.4/+0.6/−0.2°. Bias CI and LoA NR in the inspected figure.	Figure 2 signed differences. Clinical convention: >2° or >2 mm. ICCs assessed examiner/software repeatability, not AI–human agreement.
Bor 2024 [5] *n* = 120	WebCeph, WeDoCeph, CephX/NemoCeph	Calculated WebCeph minus reference for Classes I/II/III (*n* = 40 each): SNA +0.41/+0.87/+0.34°; SNB −0.91/−0.56/−1.15°; ANB +1.30/+1.40/+1.47°. WeDoCeph minus NemoCeph, Classes I/II/III: SNA −0.85/−0.15/−0.36°; SNB −0.88/−0.49/−0.65°; ANB +0.04/+0.31/+0.26°. CephX: SNA −0.38/+0.05/−0.23°; SNB −0.69/−0.39/−0.52°; ANB +0.30/+0.41/+0.29°. Source: Table 1.	Rounded group-mean differences, not MAE or LoA. Paired CI NR. Observer ICCs were separate. No numerical angular acceptability margin was defined.
Çoban 2022 [14] *n* = 105	WebCeph/Dolphin	Reference minus AI, mean (paired SD): −1.30 (2.74)/+0.15 (2.45)/−1.45 (1.46)°. ICC: 0.855 (0.786–0.901)/0.927 (0.892–0.950)/0.959 (0.940–0.972).	ICC formulation incompletely specified. Class III ANB bias −2.49°. No numerical clinical margin defined. Mean bias does not establish individual acceptability.
Duran 2023 [15] *n* = 50	OrthoDx, WebCeph/Dolphin 11.8	Dolphin minus OrthoDx, mean (paired SD): +1.33 (2.49)/+0.95 (2.23)/+0.38 (1.40)°. ICC: 0.750/0.846/0.876. Dolphin minus WebCeph: −1.03 (2.74)/+0.08 (2.28)/−1.06 (1.83)°. ICC: 0.736/0.862/0.791. Non-sagittal examples: WebCeph corpus-length difference −8.32 mm (paired SD 8.45; ICC 0.246); ramus-height difference −4.49 mm (4.34; ICC 0.358). Source: Tables 1–2, journal pp. 485–486.	Tables 1–2: direct method comparisons; Table 3: examiner comparison. ICC model/form/unit and numerical clinical margin NR. Sagittal bias CIs, ICC CIs and LoA NR.
Eldeeb 2026 [16] *n* = 57	WebCeph, Carestream V8/OnyxCeph3	OnyxCeph minus WebCeph bias (95% CI): −0.58 (−1.71 to 0.54)/+0.25 (−0.49 to 0.99)/−0.84 (−1.54 to −0.14)°. OnyxCeph minus Carestream bias (95% CI): −0.81 (−2.84 to 1.22)/−0.47 (−2.83 to 1.90)/+0.18 (−0.79 to 1.14)°. Main-text between-method ICCs: WebCeph > 0.8 except 1u–NA 0.529; Carestream 0.386–0.833 except anterior facial height 0.970. Exact SNA/SNB/ANB between-method ICCs: NA. Sources: Results, p. 5; Table 5, p. 9.	Table 5 mean-difference CIs, not LoA. Source criterion: <2°/<2 mm. Exact between-method ICCs are in a separate publisher supplement (NA). Main Table 2 contains examiner reliability, not AI–reference agreement.
Foroozandeh 2025 [17] *n* = 60	Automatic/corrected AudaxCeph; manual and Dolphin	Three-method average-measure ICCs with automatic output: 0.980 (0.969–0.988)/0.995 (0.992–0.997)/0.963 (0.935–0.978). Corrected three-method ICCs: 0.999 (0.998–0.999)/0.999 (0.999–1.000)/0.998 (0.997–0.999). Calculated automatic minus manual means +0.52/−0.15/+0.62°; corrected +0.03/+0.13/+0.03°. Sources: Tables 3–5.	Two-way random, absolute agreement, average of three methods—not a single AI–reference pair. No numerical clinical margin was prespecified.
Gupta 2024 [18] *n* = 40	WebCeph automatic/corrected/manual	Manual minus automatic, mean (paired SD): −4.45 (9.66)/−1.30 (1.80)/−1.60 (1.41)°. Manual minus corrected: −1.38 (9.26)/+0.65 (1.59)/−0.55 (1.50)°.	Four-method ICCs 0.707/0.707/0.949 are not pairwise AI–manual agreement. Bias CIs and LoA NR; no numerical clinical margin defined.
Jeon and Lee 2021 [19] *n* = 35	CephX/V-ceph	Reference minus AI bias (95% LoA): −0.5 (−4.1 to 3.1)/−0.4 (−3.3 to 2.6)/−0.1 (−2.9 to 2.7)°. Paired SDs 1.8/1.5/1.4°.	Small bias coexists with several-degree individual limits. Bias CIs NR. No a priori numerical LoA acceptability margin defined.
Köz 2025 [20] *n* = 110	WebCeph/manual	Pairwise ICC (95% CI): 0.692 (0.390–0.826)/0.883 (0.829–0.920)/0.845 (0.434–0.934). S–N distance ICC 0.215 (−0.137 to 0.473).	Separate from repeatability in 30 images. ICC model/form/unit incompletely specified. Bias, LoA and numerical clinical margins NR.
Kunz 2025 [21] *n* = 47 analysed	DentaliQ.ortho, WebCeph, AudaxCeph, CephX/expert median	WebCeph minus human bias (95% CI): +0.06 (−0.91 to 1.02)/−0.21 (−0.97 to 0.56)/+0.28 (−0.20 to 0.76)°. DentaliQ.ortho bias (95% CI): −0.11 (−0.56 to 0.33)/+0.03 (−0.38 to 0.44)/−0.13 (−0.42 to 0.17)°. AudaxCeph: +1.36 (0.81–1.91)/+1.10 (0.62–1.59)/+0.29 (0.05–0.53)°. CephX: +0.35 (−0.19 to 0.89)/+0.08 (−0.38 to 0.54)/+0.30 (0.01–0.60)°. Sources: Tables 3–4; adjusted source CIs retained.	Good average trueness: |bias| < 0.5°; good precision: SD of differences <1.5°. These are distinct criteria. Three images excluded for artefacts.
Lee 2023 [22] *n* = 84	CellmatiQ, CephX, AudaxCeph, WebCeph/expert 1	Expert minus automatic WebCeph bias (95% LoA): −1.33 (−6.71 to 4.06)/−0.24 (−4.48 to 4.00)/−1.10 (−4.01 to 1.81)°. WebCeph bias CIs: SNA −1.92 to −0.73°; SNB −0.71 to 0.23°; ANB −1.42 to −0.78°. Other automatic-platform biases (LoA): CellmatiQ −0.18 (−4.82 to 4.45)/+0.24 (−3.66 to 4.15)/−0.44 (−2.80 to 1.92)°; CephX +0.11 (−3.27 to 3.48)/+0.29 (−2.98 to 3.56)/−0.20 (−2.36 to 1.95)°; AudaxCeph −0.36 (−5.00 to 4.27)/−0.17 (−4.24 to 3.90)/−0.20 (−1.87 to 1.47)°. Source: Part 1, Table 6.	Two-way random, single-measure agreement and consistency ICCs. Human-error MRE thresholds: 1.84°/1.66°/1.21°. No automatic platform met all three sagittal interchangeability criteria.
Mahto 2022 [23] *n* = 30	WebCeph/manual	Pairwise ICC (95% CI): 0.879 (0.745–0.943)/0.899 (0.770–0.954)/0.908 (0.747–0.961).	Separate from 10-image repeatability. ICC model/form/unit incompletely specified. Mean bias, LoA and numerical clinical margin NR.
Mercier 2024 [6] *n* = 408	WebCeph automatic/corrected/NemoCeph	Automatic minus reference bias (95% CI): +1.037 (0.806–1.268)/+0.367 (0.177–0.558)/+0.660 (0.540–0.780)°. Corrected bias (95% CI): +0.062 (−0.085 to 0.209)/+0.068 (−0.055 to 0.191)/−0.018 (−0.099 to 0.062)°. L1–NB angular bias: automatic −2.414°; corrected −1.505°. Sources: Tables 3–6.	Signed bias, not MAE. Source applies 1°/1 mm and 2°/2 mm conventions separately. Excludes 25 AI-recognition failures. ICCs assess human reliability.
Meriç and Naoumova 2020 [24] *n* = 40	CephX automatic/corrected/mean of three manual observers	Calculated automatic minus manual: +0.1/0.0/0.0°; corrected: +0.2/+0.1/+0.1°. Paired CI and LoA NR.	Source uses a 2-unit convention. GoGn–SN difference 6.5° automatic and 4.9° corrected, with differing mandibular-plane definitions. Repeatability ICCs are separate.
Raby 2025 [25] *n* = 50	CephX and WebCeph, automatic/corrected/Dolphin 13.01	SNA/SNB/ANB ICCs: CephX automatic 0.93/0.96/0.95; corrected 0.92/0.95/0.98. WebCeph automatic 0.81/0.91/0.83; corrected 0.93/0.96/0.97. Calculated AI minus Dolphin means: CephX automatic +0.06/+0.51/−0.42°; corrected +0.27/+0.28/−0.05°; WebCeph automatic +0.86/+1.11/−0.28°; corrected +0.01/+0.26/−0.29°. The one printed ICC CI is 0.83–0.91 for SNA and 0.87–0.93 for SNB/ANB; it cannot be assigned to individual arms. Sources: Tables II and IV, pp. 509–510. These calculated mean differences are not individual LoA.	Table IV uses Dolphin as ground truth; calibration ICC 0.994 is separate. One CI column is not assigned to individual arms. ICC model/form/unit and numerical LoA NR. Individual ±2°/±2 mm assessment differs from comparison of means; SNA/SNB supported only after correction.
Tsolakis 2022 [26] *n* = 100	CS Imaging V8/Dolphin	Pairwise ICC (95% CI): 0.74 (0.55–0.90)/0.78 (0.60–0.94)/0.85 (0.70–0.97). Calculated automatic minus manual means: +0.7/+0.7/+0.2°.	ICC formulation incompletely specified. Bias CIs and LoA NR. Non-significant tests are not equivalence tests; no numerical clinical margin defined.
Yassir 2022 [27] *n* = 50	WebCeph automatic/corrected/AutoCAD	Automatic minus reference bias (95% CI): +0.15 (−0.59 to 0.89)/−1.29 (−1.90 to −0.67)/+1.45 (1.10–1.80)°. Corrected bias (95% CI): −0.07 (−0.35 to 0.20)/−0.02 (−0.24 to 0.21)/−0.02 (−0.19 to 0.16)°. Automatic absolute-agreement ICCs: 0.828 (0.696–0.902)/0.828 (0.597–0.916)/0.825 (0.049–0.942). L1–MP bias remains −7.42° automatic and −8.42° corrected. Sources: Tables 2–3.	Pairwise single-measure absolute-agreement ICCs. Clinical difference >2°/>2 mm. Proportional bias for SNA/SNB; mean-bias CIs are not LoA.
Zaheer 2024 [28] *n* = 54	CephX automatic; WebCeph semi-automatic/manual	Calculated CephX minus manual for examiners 1/2: SNA +0.26/+0.70°; SNB +0.51/+0.27°. ANB was not measured. WebCeph semi-automatic minus manual, examiners 1/2: SNA +1.51/+1.32°; SNB +1.35/+0.91°. Source: Table 1; calculated from rounded means.	ICC compares examiners within methods, not AI versus manual. Paired CIs and LoA NR; no numerical clinical margin defined. Examiners are not separate cohorts.
Zughair 2025 [29] *n* = 120	WebCeph automatic; semi-automatic workflow/OneCeph manual	Calculated automatic minus manual: +1.29/+0.31/+0.96°; semi-automatic: +0.08/+0.12/+0.05°. Paired CIs and LoA NR.	WebCeph version not reported. No explicit clinical threshold applied. Group-mean differences and significance tests do not establish individual agreement.

Values separated by slashes refer to SNA/SNB/ANB, in that order, unless a different ordering is stated. Angular differences are in degrees. Confidence intervals are 95% unless stated otherwise. NR, not reported in the identified source; NA, not available for extraction. Signed mean differences, mean absolute errors, and limits of agreement are not interchangeable. Multiple software arms use the same study sample and are not independent studies.

## Data Availability

All data analysed are included in this study. Further enquiries are available upon request from the corresponding author.

## Supplementary Materials

The following supporting information can be downloaded at: https://www.mdpi.com/article/10.3390/tomography12100146/s1.
