# Supplementary material for: Beyond Landmark Error: Agreement Between AI-Assisted and Manual Cephalometric Tracing at the Level of Clinical Measurements: A Systematic Review
Source: Tomography. 2026 Oct 2;12(10):146. doi: 10.3390/tomography12100146 (PMC13709440; doi:10.3390/tomography12100146)
Supplement: Supplementary file 1 [file tomography-12-00146-s001.zip › tomography-4590171-supplementary(2).pdf]

Supplementary Table S1. Studies excluded during detailed full-text assessment, with reasons for exclusion.

No	Study (author, year)	Reference	Reason for exclusion
1	Zecca et al., 2025	[32]	AI prediction of cephalometric values (reduced-field prediction of missing values), not AI-versus-manual/expert tracing agreement
2	Fracchia et al., 2025	[33]	Single-radiograph reproducibility (preliminary study) without a patient-level AI-versus-reference diagnostic comparison
3	Lin et al., 2025	[34]	Educational intervention evaluating student landmarking accuracy, not an AI-versus-manual/expert tracing comparison
4	Lee et al., 2023 (Part 2)	[35]	Companion report (Part 2) analysing the same 84-cephalogram sample already represented by the included Lee et al. Part 1 [20]; excluded to avoid duplicate use of the same cohort
5	Panesar et al., 2023	[36]	Reported intraclass correlation coefficients reflect intra-rater precision (test–retest reproducibility); agreement with the gold standard was expressed as mean absolute error rather than an AI-versus-manual/expert ICC, so no poolable agreement estimate was available
